# Evaluation of Different Extraction Methods on the Phenolic Profile and the Antioxidant Potential of *Ceratonia siliqua* L. Pods Extracts

**DOI:** 10.3390/molecules27196163

**Published:** 2022-09-20

**Authors:** Fouad El Mansouri, Joaquim C. G. Esteves Silva, Francesco Cacciola, Fadoua Asraoui, Hatim Tayeq, Yasmine Mttougui Ben Amar, Miguel Palma Lovillo, Noureddine Chouaibi, Jamal Brigui

**Affiliations:** 1 Research Team: Materials, Environment and Sustainable Development (MEDD), Faculty of Sciences and Techniques of Tangier, Abdelmalek Essaâdi University, Tangier BP 416, Morocco; 2 Centro de Investigação em Química (CIQUP), Instituto de Ciências Moleculares (IMS), Departamento de Geociências, Ambiente e Ordenamento do Território, Faculdade de Ciências, Universidade do Porto, Rua do Campo Alegre s/n, 4169-007 Porto, Portugal; 3 Department of Biomedical Dental Morphological and Functional Imaging Sciences, University of Messina, 98125 Messina, Italy; 4 Laboratory of Applied Biology and Pathologies, Department of Biology, Faculty of Sciences of Tetouan, Abdelmalek Essaâdi University, Tetouan 93000, Morocco; 5 MAE2D Laboratory, Polydisciplinary Faculty of Larache, Abdelmalek Essaâdi University, Tetouan 93000, Morocco; 6 Department of Analytical Chemistry, Faculty of Sciences, Instituto de Investigación Vitivinícola y Agroalimentaria (IVAGRO), University of Cadiz, Campus del Rio San Pedro, 11510 Cadiz, Spain; 7 Laboratory of LAMSE, Faculty of Sciences and Techniques of Tangier, Tangier BP 416, Morocco

**Keywords:** *Ceratonia siliqua* L., pods, polyphenols, flavonoids, tannins, antioxidant activity, extraction methods, HPLC

## Abstract

The present work was designed to investigate the effects of different extraction processes, namely ultrasonic-assisted, supercritical fluid, microwave-assisted and Soxhlet applied to carob pods. The total phenolic quantification and the antioxidant activity were assessed by the means of rapid in vitro spectrophotometric assays; the phenolic profile was identified using ultra-high performance liquid chromatography coupled to mass spectrometry. The results revealed that the phenolic compounds and the antioxidant capacity varied significantly with the nature of the extraction process. The content of total phenolic compounds ranged from 11.55 to 34.38 mg GAE/g DW; the content of total flavonoids varied from 3.50 to 10.53 mg QE/g DW, and the content of condensed tannins fluctuated from 3.30 to 6.55 mg CE/ g DW. All extracts performed differently on antioxidant activity when determined by the DPPH assay producing a dose-dependent response, with IC_50_ extended from 11.33 to 6.07 µg/mL. HPLC analysis enabled the identification of nine compounds. As a function of the studied extraction methods, the phenolic compound contents were positively correlated with antioxidant activity.

## 1. Introduction

Carob (*Ceratonia siliqua* L.) is an evergreen tree belonging to the Fabaceae (legume) family, cultivated or naturally grown in the Mediterranean region and the Middle East [1]. The carob tree, *Ceratonia siliqua* L., is well-known for its interesting medicinal properties. In fact, due to its chemical composition, carob is used in the food industry and medicine. In terms of medicinal uses, it has been revealed that carob pods exert anti-inflammatory, antimicrobial, anti-diarrheal, anti-ulcer, anti-constipation, and anti-absorptive of glucose activities in the gastrointestinal tract [2,3,4]. Moreover, the leaves and the bark of carob were described as having an excellent effect when used as antidiabetic agents [5]. As already described for several medicinal plants [6,7,8], in vitro, and in vivo studies of the antioxidant capacities of carob proved their inhibiting potential, related to their phenolic compounds [9,10,11]; moreover, carob has a great potential to be used in agri-food industries due to its phytochemical constituents with functional, flavoring properties through its characteristic strong aroma, which persists even after treatment [12]. This exceptional quality may be clarified by the presence of acids, esters, and aldehydes/ketones produced from carob fruit and powder, which are biogenic volatile organic composites that promote plant growth, breeding, protection [13,14] and nutrition benefits [15]. In addition, it has been confirmed to possess remarkable bioactivity and is considered dietary fiber in the food industry [16,17], wherein it is used for the preparation of soft drinks, confectionery products, and baked goods and as a substitute for cocoa or chocolate [18]. Furthermore, a variety of bioactive compounds were found in all parts of *Ceratonia siliqua* L., (leaves, pods, and seeds), such as phenolic acids, flavonoids, tannins, and alkaloids, as well as nutritional compounds, such as vitamins, protein, lipids, and minerals. [19,20].

Reactive oxygen species (ROS) can play a crucial physiological function or a toxic effect, depending on their concentrations in the human body, during the normal physiological state. ROS are produced in minor concentrations to control apoptosis or activate transcription aspects, acting as a messenger in the intercellular signalling pathway. The high production of ROS turns out to be pathologic and toxic through the activation of the gene expression coding for proinflammatory cytokines. Their unbalanced state made them extremely reactive about biological substrates; generated damaging oxidative alterations; and leads to oxidative stress implicated in various pathologies, especially in cancer engendered by DNA mutations [21], The high production of ROS in the cell provokes cellular ageing and apoptosis, resulting in the altered redox regulation of intracellular signaling cascades implicated in several diseases including diabetes, rheumatoid arthritis, cancer, neurodegenerative diseases, and atherosclerosis, [22] Indeed, antioxidants are a group of compounds able to react with free radicals by neutralizing them to non-radical products; therefore, such compounds can be used to block or minimize the damaging impacts caused by free radicals in the human body and contribute to maintaining the balance between free radicals produced and antioxidants [23]. Phenolic compounds are metabolites resulting from L-phenylalanine, containing a large group of substances such as phenolic acids, hydroxycinnamic acids, lignans, tannins, and flavonoids, and they have shown great beneficial effects in terms of pharmacological uses [24,25].

The present work aims to highlight the effect of different extraction methods for carob pods on the concentration of phenolic compounds, along with an evaluation of the antioxidant potential via in vitro spectrophotometric assays of the different obtained extracts; in addition, the phytochemical profile was performed by high-performance liquid chromatography (HPLC).

## 2. Material and Methods

### 2.1. Plant Material

Fresh carob fruits (*Ceratonia siliqua* L.) were harvested from the northwest Morocco Taoughilte area (34°24′37.4″ N, 5°29′12.8″ W) during ripening (ripe stage). Identification was confirmed, and they were placed in the herbarium of the laboratory with the voucher specimen code CS-LCEVR02. The seeds were manually removed after fruit drying in the dark at ambient temperature. The pods were ground in an electrical grinder to obtain a fine powder, sieved through 100 mesh stainless steel sieves, and stored at 4 °C for further analysis.

### 2.2. Extraction Procedures

#### 2.2.1. Ultrasound-Assisted Extraction (UAE)

Ultrasound-assisted extraction (UAE) was applied for the extraction of phenolic compounds from ripe carob pods: for this purpose, acetone was chosen as a solvent, and 10 g of carob pulp powder was mixed with an appropriate volume of acetone solution with a solid–solvent ratio. The tip of the probe was submerged 2 cm into the extraction solution, and the sonication was conducted in both continuous (0s:0s) and pulsed (5s:5s) modes. The temperature of the ultrasonic water bath was regulated to 50 °C. The extraction took 30 min and was repeated 3 times. The power of the sonicator used in our tests was 400 W. The achieved mixture was exposed to centrifugation at 4400 rpm for 20 min; the solution was filtered through Whatman filter paper No.1, and then the sample was concentrated under vacuum using a rotary evaporator (RE300, stuart) and stored at −20 °C until further analysis.

#### 2.2.2. Microwave-Assisted Extraction (MAE)

The microwave-assisted extraction (MAE) (Ethos 1600 microwave extractor, Milestone, Shelton, CT, USA) of carob pulp powder was performed under the following extraction conditions in a closed vessel: temperature: 100 °C; solvent: acetone/water (57/43, *v/v*); power: 400 W; magnetic stirring: 0–100%. A sample of powdered pulp (10–50 mg. mL^−1^) was mixed with 15–20 mL of solvent for 5 to 20 min; the heating of the samples was assisted only by microwave energy.

#### 2.2.3. Soxhlet Extraction

The Soxhlet extraction of carob pod powder was conducted using a Soxhlet apparatus with 150 mL of aqueous acetone solution (57%, *v/v*, acetone) for 6 h, then the organic phase was concentrated and made free of solvent under reduced pressure, using a rotary evaporator, then evaporated to dryness, and the residual aqueous phase was frozen and lyophilized; the obtained extracted was stored at −20 °C until further analysis.

#### 2.2.4. Supercritical Fluid Extraction (SFE-CO_2_)

Supercritical carbon dioxide extraction (SFE-CO_2_) was achieved in a Helix extraction system (Applied Separation, Allentown, PA, USA) with 99.9% CO_2_ in agreement with a procedure earlier established in [26]. Dry ground material was deposited in a 50 cm cylindrical extractor (14 mm inner diameter and 320 mm length). Cotton wool was placed at the top and bottom of the extraction vessel. In all extractions, the CO_2_ flow rate was kept constant at 2 L/min (standard conditions). The extraction time was 180 min (including 30 min of static extraction time); the pressure was 45 MPa, and the temperature was 70 °C. The extracts were stored in glass vials and kept refrigerated at 4 °C until further handling.

### 2.3. Analysis and Quantification of Phenolic Contents

#### 2.3.1. Total Phenol Content (TPC)

An assessment of the total phenolics of the extracts was evaluated through a spectrophotometric method using the Folin–Ciocalteu reagent according to the procedure described by [27] and slightly modified by [28]; for this aim, 100 µL of diluted sample was mixed with 500 µL of Folin–Ciocalteu reagent solution (10%); thereafter, the combination was basified by adding 400 mL of 7.5% sodium carbonate (Na_2_CO_3_); then, the solution was shaken thoroughly, then allowed to stand in the dark for 60 min at room temperature, and the absorbance was measured at 765 nm. The TPC in the samples was estimated from a calibration curve prepared with gallic acid as a standard with different concentrations and expressed as mg of gallic acid equivalents (GAE) per g of dry weight (mg GAE/g DM) of the sample. Tests were done in triplicate, and the results are given as the mean average.

#### 2.3.2. Total Flavonoid Content (TFC)

The flavonoid contents were achieved according to the protocol described by [29] This colorimetric method consists of homogenizing 250 µL of a diluted sample with 1 mL of AlCl_3_ solution (2%). The samples were incubated for 1 h at room temperature. The absorbance was determined using a spectrophotometer at λ_max_ = 415 nm. The total flavonoids were expressed as quercetin equivalents by reference to the quercetin standard calibration curve (mg QE/g DM). Tests were done in triplicate, and the results are given as the mean average.

#### 2.3.3. Condensed Tannins Content (CTC)

The condensed tannins were determined by the following method [30]. Thus, 0.5 μL of each sample was added to 3 mL of vanillin reagent (4% of methanolic vanillin) and 1.5 mL of hydrochloric acid. Then, the solution was allowed to stand at room temperature for 15 min. This method is based on the ability of vanillin to react with condensed tannins in the presence of acid to produce a colored complex measured at 500 nm. The results were expressed as mg catechin equivalents per g of dry mass. Tests were done in triplicate, and the results are given as the mean average.

### 2.4. Antioxidant Capacity

#### Free Radical Scavenging Assay

The antioxidant capacity of carob extracts was assessed by 2,2-diphenyl-1-picrylhydrazyl radical (DPPH) according to the procedure of [31]; for this purpose, different concentrations of carob extracts (50–200 µg) were diluted in 3 mL of methanol and mixed with 3 mL of the DPPH ethanol solution (200 µM). The solution was mixed thoroughly, then incubated in the dark at room temperature for 30 min. The absorbance of ethanol was considered as the blank and measured at 517 nm. The percentage of antioxidant activity at different concentrations was determined by using ascorbic acid as the reference standard. The % free radical scavenging activity was calculated using the given equation:

% Antioxidant activity = (A_control_ − A_extract_/A_control_) × 100

where A_control_: Absorbance of control; A_extract_: Absorbance of extract.

The percentage of antioxidant activity versus the concentration of the extract was plotted. The IC_50_ value (μg/mL) was obtained by interpolation from the logarithmic regression analysis.

### 2.5. High-Performance Liquid Chromatography Analysis

A Beckman HPLC system was employed for the detection of phenolic compounds in carob pod extracts (Fullerton, CA, USA); a Discovery RP-C18 reversed-phase column (Supelco, 250 mm × 4.6 mm; 5 μm d.p.) was used as an analytical column. Compounds were separated using the solvent gradient A (Water/formic acid; 19/1) and B (Methanol) described by [18] and detected at 280 nm with a UV detector (D166). The chromatograms were analyzed by Gold Analysis v1.5 software (Beckman Instruments, CA, USA). The content of the identified phenolic compounds was calculated by correlating the measured peaks with the calibration curves obtained by reference compounds (provided by Sigma-Aldrich, Paris, France), namely gallic acid, protocatechic acid, 4-hydroxy-benzoic acid, 4-hydroxy-acetic acid, vanillic acid, syringic acid, p-coumaric acid, m-coumaric acid, coumaric acid, ferulic acid, benzoic acid, oleuropein acid, and hydroxytyrosol).

### 2.6. Statistical Analysis

Results are reported as the means of triplicate analysis. Data obtained were subjected to a one-way analysis of variance (ANOVA) for assessing the significance of quantitative changes in the variables as a result of the different extraction methods. The statistical analysis was done by the Statistical Package for Social Science (SPSS 26.0).

## 3. Results and Discussion

### 3.1. Phytochemical Analysis of Carob

Phenolic compounds are considered natural antioxidants and vital biological compounds in plant materials; these composites exhibit a crucial role in preventing certain diseases, including disorders related to reactive oxygen species [32] and could be extracted from plant material using different organic solvents using different extraction methods. For this reason, four commonly extraction methods were used in this study, using acetone 70% as a solvent for the extraction process; acetone has been reported to be a suitable solvent for extracting phenolic compounds from different plant materials. This is due to the solubility of some crucial bioactive compounds of polyphenols in such a solvent [30,33,34,35].

**Figure 1:**
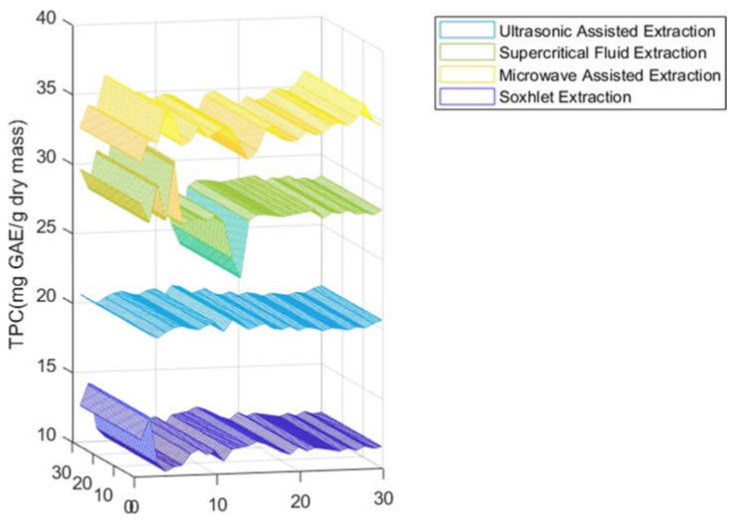
TPC of carob pod extracts using different extraction methods.

**Figure 2:**
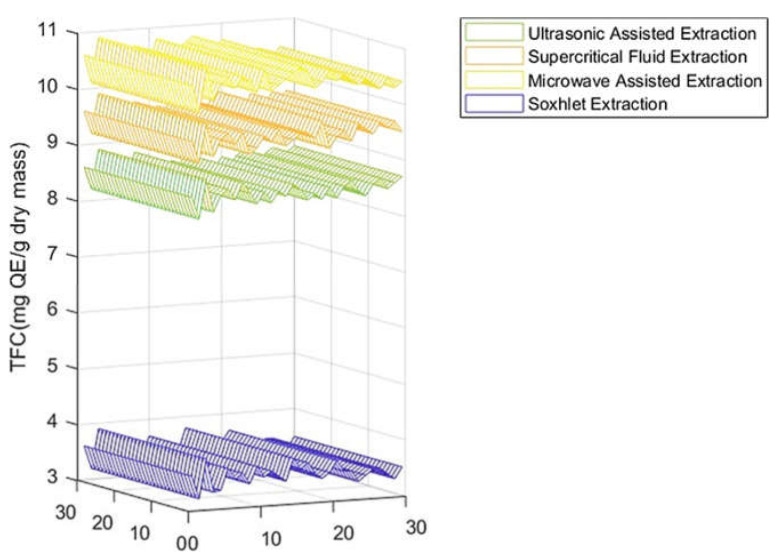
TFC of carob pod extracts using different extraction methods.

**Figure 3:**
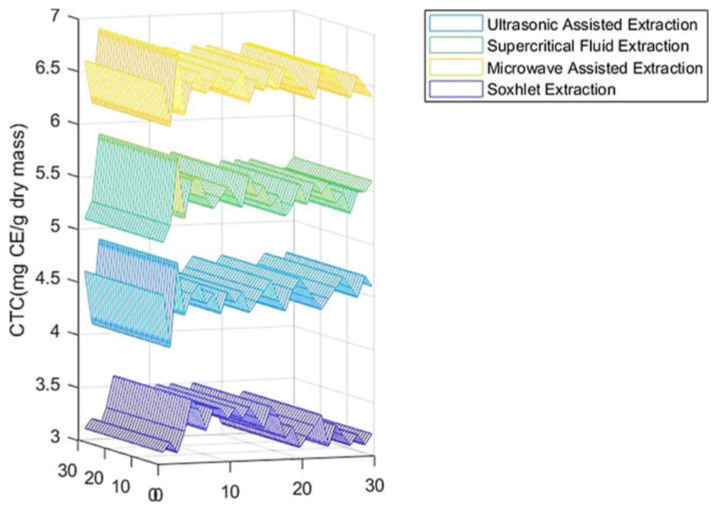
CTC of carob pod extracts using different extraction methods.

As shown in Figure 1 and Table 1, the highest amount of total phenolic content was found in the MAE extract with 34.35 mg GAE/g DM, followed by the SFE-CO_2_ extract with a value of 28.38 mg GAE/ g DM, and values of 20.38 mg GAE/g DM, and 11.55 mg GAE/g DM were attained for the UAE and Soxhlet extracts, respectively. On other hand, the same observation was made for the total flavonoid content of the carob extracts revealed to be strongly affected by the extraction process (Figure 2, Table 1), varying between 3.50 and 10.53 mg QE/g DM. MAE turned out to be the most appropriate extraction method for the recovery of carob flavonoids, yielding the highest value (10.53 mg QE/g DM). For the condensed tannins (proanthocyanidins), the amounts calculated were 6.55 mg CE/g DM, 5.49 mg CE/g DM, 4.55 mg CE/g DM, and 3.30 mg CE/g DM for MAE, SFE-CO_2_, UAE, and soxlet, respectively (Figure 3, Table 1). The results achieved in these studies are in agreement with previous reports concerning total phenols, flavonoids, and condensed tannins [36]. According to other studies, carob pods might show variation, depending on the solvent used during the extraction process [37]. As previously reported [38,39,40], MAE can be considered a suitable and fast extraction process wherein microwave energy is delivered efficiently to materials through molecular interaction with the electromagnetic field and offers a rapid transfer of energy to the extraction solvent and raw plant materials [41,42].

### 3.2. Antioxidant Capacity (DPPH Assay)

**Figure 4:**
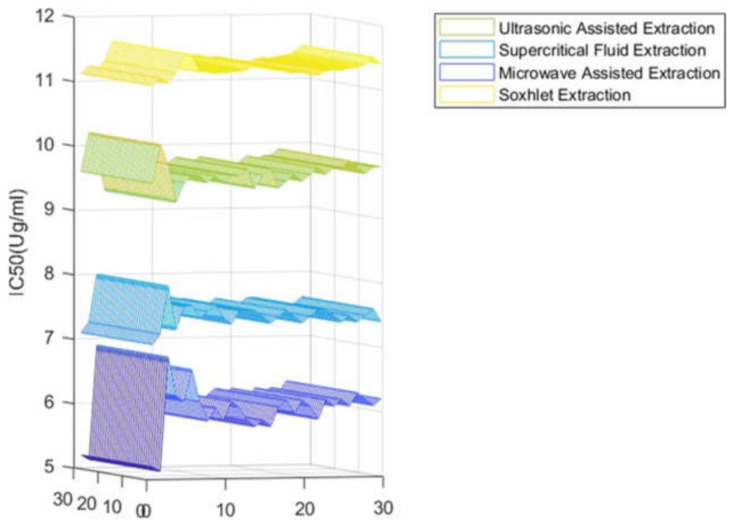
Antioxidant activity of carob pod extracts using different extraction methods.

The four extracts were further investigated using the DPPH assay to evaluate the antioxidant activity of carob extracts. Data are summarized in Figure 4 and Table 1, wherein it can be appreciated how the antiradical potential of carob samples increases in a dose-dependent manner. This antioxidant ability can be assessed by the determination of IC_50_ values related to the amount of the sample required to reduce 50% of free radicals. The most effective antioxidant extract was found to be the MAE extract with an IC_50_ of 6.07 µg/mL, which had the highest concentration of phenolic compounds (34.38 mg GAE/g DW), followed by SFE-CO_2_, UAE, and Soxhlet extracts with values of IC_50_ = 7.51 µg/mL, 9.71 µg/mL, and 11.33 µg/mL, respectively.

Consequently, carob pods show a significant antioxidant capacity, probably due to the levels of total phenols, flavonoids, and condensed tannins. In addition, phenolic compounds, depending on their different characteristics, contribute to the antioxidant propriety in a dose-dependent manner until a maximum of activity [43]. The results attained in this work are consistent with previous findings [34,44,45].

**Figure 5:**
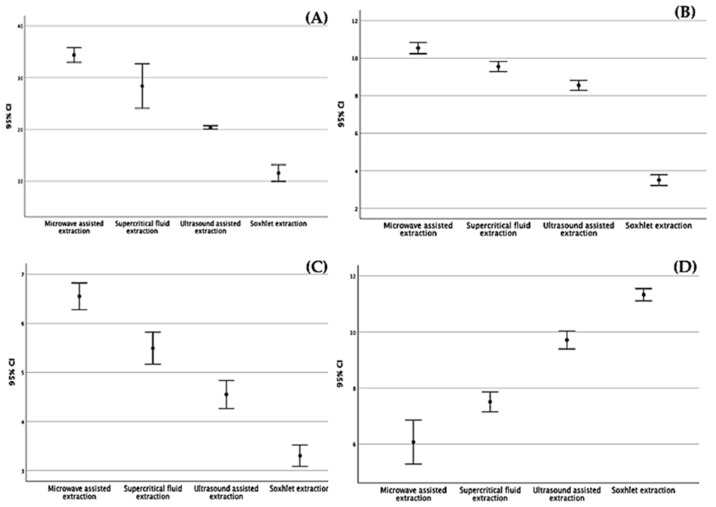
The 95% confidence intervals for the main analysis of (**A**) total phenols, (**B**) total flavonoids, (**C**) condensed tannins, and (**D**) antioxidant activity using different extraction methods.

The 95% confidence intervals for the main analysis of total phenol, total flavonoid, condensed tannin content, and antioxidant activity using different extraction methods are illustrated in Figure 5.

### 3.3. Phenolic Constituents of Carob Extracts by HPLC

**Figure 6:**
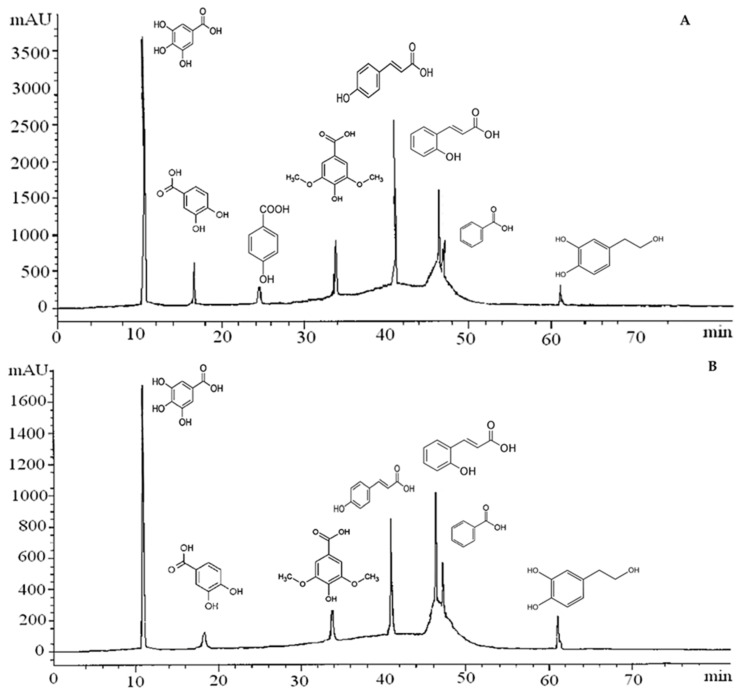

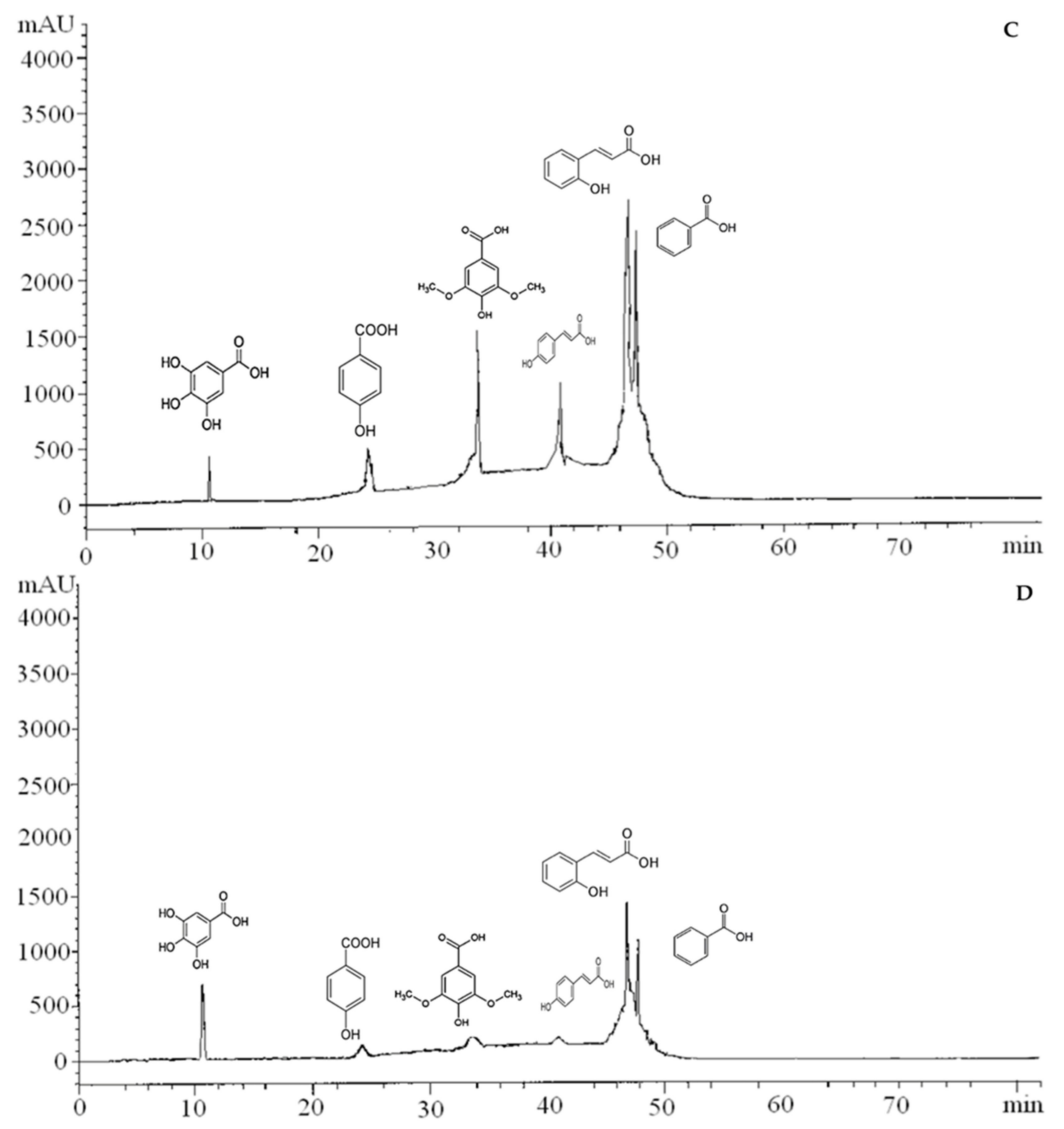
Chromatograms of detected phenolic compounds by the different extraction methods, (**A**) microwave-assisted extraction, (**B**) supercritical fluid extraction, (**C**) ultrasound-assisted extraction, (**D**) Soxhlet extraction.

The carob pod extracts were subjected to HPLC analysis, and the phenolics compounds attained are displayed in Figure 6 and Table 2, which reveal significant quantitative and qualitative variation among the studied extracts. It was found that the MAE extract is richer in phenolic compounds in comparison with the other extracts. Coumaric acid was the main phenolic compound in all extracts with a content ranging from 8.18% to 20.05%. Furthermore, the MAE and the SFE-CO_2_ extracts contained a significant amount of gallic acid, 18.57% and 17.80%, respectively. Protocatechuic acid and hydroxytyrosol were detected only on MAE and SFE extracts in a low amount. In general, the phenolic profile could, at least in part, justify the significant antioxidant activity, especially for MAE extract, and almost the totality of this substance has been reported in the literature [10,46,47,48].

The statistical analysis reported in Table 3 shows a significant difference (*p* < 0.05) between the means obtained by each type of different extraction methods in all of the assays studied.

## 4. Conclusions

The overall objective of this work was to carry out a direct comparison of four extraction processes, namely microwave-assisted, supercritical fluid, ultrasonic-assisted, and Soxhlet. The efficiency of the extraction was determined by considering the total phenolic compounds concentration. To this regard, the highest amount of total phenolic content was found in the microwave-assisted extraction with 34.38 mg GAE/g DM, followed by the supercritical fluid extraction with a value of 28.38 mg GAE/g DM. Likewise, also for total flavonoid and condensed tannin contents, the microwave-assisted extraction turned out to be the most effective one (10.53 mg QE/g DM; 6.55 ± 0.23 mg CE/g DM). With regards to antioxidant activity, a dose-dependent response with IC_50_ extended from 6.07 to 11.33 to µg/mL was achieved.

According to the results of the present study, microwave-assisted extraction might be considered a promising alternative compared to the others for the recovery of bioactive compounds from plant materials. Notably, microwave-assisted extraction is less power-demanding (and hence less expensive). Future research should be focused on the optimization of the process by using different microwave-assisted extraction parameters (extraction time, power, and magnetic stirring speed), in order to optimize and increase the extraction efficiency.

## Figures and Tables

**Table 1:** TPC, TFC, CTC, and antioxidant activity of carob pod extracts.

Extraction Methods	TPC (mg GAE/g Dry Mass)	TFC (mg QE/g Dry Mass)	CTC (mg CE/g Dry Mass)	DPPH IC_50_ Value (µg/mL)
Microwave-assisted extraction	34.35 ± 1.22	10.53 ± 0.26	6.55 ± 0.23	6.07 ± 0.68
Supercritical fluid extraction	28.38 ± 3.75	9.55 ± 0.23	5.49 ± 0.28	7.51 ± 0.30
Ultrasound-assisted extraction	20.38 ± 0.27	8.55 ± 0.23	4.55 ± 0.25	9.71 ± 0.27
Soxhlet extraction	11.55 ± 1.40	3.50 ± 0.24	3.30 ± 0.18	11.33 ± 0.19

**Table 2:** Comparison of phenolic compounds identified carob pods with different extraction methods.

	% of Detected Phenolic Compounds			
	SFE-CO_2_	UAE	MAE	Soxhlet
Gallic acid	17.80 ± 9.06	6.01 ± 3.42	18.57 ± 5.67	3.95 ± 2.09
Syringic acid	3.24 ± 2.45	3.82 ± 0.63	5.11 ± 3.18	4.12 ± 1.23
Coumaric acid	17.52 ± 8.76	9.07 ± 5.79	20.05 ± 10.20	8.18 ± 7.26
*p*-coumaric acid	10.78 ± 6.27	7.47 ± 3.23	13.27 ± 4.15	6.55 ± 3.86
*m*-coumaric acid	9.73 ± 5.56	2.6 ± 1.37	10.78 ± 2.10	1.62 ± 1.31
Benzoic acid	4 ± 1.98	1.93 ± 0.93	4.19 ± 2.33	1.93 ± 0. 14
4-hydroxybenzoic acid	0.00	0.21 ± 0.11	0.23 ± 0.2	0.11 ± 0.01
Protocatechuic acid	0.13 ± 0.01	0.00	0.79 ± 0.55	0.00
Hydroxytyrosol	0.64 ± 0.62	0.00	1.21 ± 0.12	0.00

**Table 3:** Statistical analysis of the means performed by one-way analysis of variance (ANOVA).

Type of Analysis	Extraction Methods	Mean	Std. Error	95% Confidence Interval		Test ANOVA	
				Lower Bound	Upper Bound	Variance	Sig.
Total phenol content (mg GAE/g dry mass)	UAE	20.38	0.124	19.90	20.70	0.094	0.001 S
	SFE-CO_2_	28.39	1.679	20.70	32.60	16.933	
	MAE	34.38	0.549	32.60	36.30	1.814	
	Soxhlet	11.55	0.626	10.30	14.21	2.358	
Total flavonoid content (mg QE/g dry mass)	UAE	8.55	0.104	8.20	8.90	0.066	0.001 S
	SFE-CO_2_	9.55	0.104	9.20	9.90	0.066	
	MAE	10.53	0.116	10.10	10.90	0.082	
	Soxhlet	3.50	0.111	3.20	3.90	0.075	
Condensed tannins content (mg CE/g dry mass)	UAE	4.55	0.111	4.10	4.90	0.075	0.001 S
	SFE-CO_2_	5.49	0.126	5.10	5.90	0.096	
	MAE	6.55	0.105	6.20	6.9	0.067	
	Soxhlet	3.30	0.084	3.10	3.600	0.043	
DPPH IC_50_ value (μg/mL)	UAE	9.72	0.124	9.30	10.20	0.094	0.001 S
	SFE-CO_2_	7.51	0.138	7.10	8.00	0.115	
	MAE	6.07	0.305	5.13	6.90	0.560	
	Soxhlet	11.33	0.086	11.12	11.61	0.045	

Values are averages ± standard deviation of triplicate analysis. Data obtained were subjected to a one-way analysis of variance (ANOVA). S: significant (*p* < 0.05).

## Data Availability

Not applicable.
